# Highly Conserved Testicular Localization of Claudin-11 in Normal and Impaired Spermatogenesis

**DOI:** 10.1371/journal.pone.0160349

**Published:** 2016-08-03

**Authors:** Angelika Stammler, Benjamin Udo Lüftner, Sabine Kliesch, Wolfgang Weidner, Martin Bergmann, Ralf Middendorff, Lutz Konrad

**Affiliations:** 1 Justus-Liebig-University Giessen, Institute of Anatomy and Cell Biology, Aulweg 123, D-35392, Giessen, Germany; 2 Justus-Liebig-University Giessen, Department of Obstetrics and Gynecology, Feulgenstraße 12, D-35392, Giessen, Germany; 3 University Hospital Münster, Department of Clinical Andrology, Centre of Reproductive Medicine and Andrology, Domagkstrasse 11, D-48129, Münster, Germany; 4 Justus-Liebig-University / UKGM Giessen, Department of Urology, Pediatric Urology and Andrology, D-35392 Giessen, Germany; 5 Justus-Liebig-University Giessen, Institute of Veterinary Anatomy, Histology and Embryology, Frankfurter Straße 98, D-35392, Giessen, Germany; Universitatsklinikum Hamburg-Eppendorf, GERMANY

## Abstract

In this study we tested expression of tight junction proteins in human, mouse and rat and analyzed the localization of claudin-11 in testis of patients with normal and impaired spermatogenesis. Recent concepts generated in mice suggest that the stage-specifically expressed claudin-3 acts as a basal barrier, sealing the seminiferous epithelium during migration of spermatocytes. Corresponding mechanisms have never been demonstrated in humans. Testicular biopsies (n = 103) from five distinct groups were analyzed: normal spermatogenesis (NSP, n = 28), hypospermatogenesis (Hyp, n = 24), maturation arrest at the level of primary spermatocytes (MA, n = 24), Sertoli cell only syndrome (SCO, n = 19), and spermatogonial arrest (SGA, n = 8). Protein expression of claudin-3, -11 and occludin was analyzed. Human, mice and rat testis robustly express claudin-11 protein. Occludin was detected in mouse and rat and claudin-3 was found only in mice. Thus, we selected claudin-11 for further analysis of localization. In NSP, claudin-11 is located at Sertoli-Sertoli junctions and in Sertoli cell contacts towards spermatogonia. Typically, claudin-11 patches do not reach the basal membrane, unless flanked by the Sertoli cell body or patches between two Sertoli cell bodies. The amount of basal claudin-11 patches was found to be increased in impaired spermatogenesis. Only claudin-11 is expressed in all three species examined. The claudin-11 pattern is robust in man with impaired spermatogenesis, but the proportion of localization is altered in SCO and MA. We conclude that claudin-11 might represent the essential component of the BTB in human.

## Introduction

The blood-testis-barrier (BTB) was first discovered by tracer diffusion experiments [1]. Stagnation of tracer diffusion beyond the level of spermatogonia indicated the division of the seminiferous tubules into a basal and an adluminal compartment. The basal compartment is the niche for renewal and proliferation of spermatogonia whereas the adluminal compartment is the site of meiosis, and subsequently of spermiation [2, 3]. The BTB was first described to be generated by expression of the tight junction protein claudin-11 (synonym: oligodendrocyte-specific protein, OSP), which is located at the border of the basal and adluminal compartment [4, 5]. Developing spermatocytes have to cross this border [2], which is accompanied by the adluminal shift of claudin-11 and its eventual dissolution [6]. During this process, migrating spermatocytes are shielded from the basal side as demonstrated by tracer experiments [7, 8]. Whereas claudin-11 and occludin are expressed in Sertoli cells during the entire cycle of the seminiferous epithelium in mice, claudin-3 was described to be strictly stage-specific and androgen-dependent [9, 10]. According to recent findings in mice, migrating spermatocytes are embedded between adluminal “old tight junctions” containing mainly claudin-11, and basal “new tight junctions”, formed mainly by claudin-3 (synonym: rat ventral prostate protein 1, RVP-1) [6, 11]. However, these assumptions have never been investigated in human or in any other species but mouse.

Concordant with mice and rat [12–15] human testis comprises high levels of claudin-11 [16, 17]. In human testis it is controversial, whether occludin and claudin-3 are present or not [4, 18]. Also in rat, expression of claudin-3 in Sertoli cells has been questioned [19]. Expression profiles of tight junction proteins of the BTB in human have never been investigated systematically in comparison to murine and rodent testis. In this study we address the following questions: (i) Supposed there is a basal component in the human BTB, is it mediated by claudin-3 as described in mice? (ii) Can putative alterations in the basal barrier be correlated to impaired spermatogenesis? (iii) Is the tight junction protein localization altered in patients with impaired spermatogenesis?

## Material and Methods

### Patients and ethics

Written consent was obtained from each individual patient. Patients were informed and consented by signing a standardized form. The ethics committee approved the procedure (case number: paraffin embedded testicular tissue samples 75/00 and 56/05, frozen lung tissue samples AZ 31/93, approved by the Ethics Committee of the Medical Faculty of the Justus-Liebig-University, Giessen; frozen testicular tissue samples approved by Ärztekammer Hamburg, Germany, 1996). Testicular biopsy samples from 100 patients were obtained from a local tissue bank. Biopsy of testis was indicated because of normogonadotropic obstructive or non-obstructive azoospermia. Specimens were fixed in Bouin’s solution and embedded in paraffin. After staining 5 μm sections with hematoxylin and eosin, spermatogenesis was histologically evaluated applying the modified Holstein scoring system according to [20]. The patients were classified into histologically normal spermatogenesis (NSP, n = 25; median age 37, range 27–56; mean score 9.9, range 9–10), hypospermatogenesis (Hyp, n = 24; median age 37, range 26–47; mean score 8.6, range 6–10), maturation arrest at the level of spermatocytes (MA, n = 24; median age 32, range 17–47; all score 0), Sertoli cell only syndrome (SCO, n = 19; median age 35, range 21–42; all score 0), and spermatogonial arrest (SGA, n = 8; median age 38, range 31–82; all score 0), details are given in S1 Table. Cryopreserved testicular material of three patients undergoing orchiectomy for suppression of androgen (n = 3; age 65, 77 and 31), whose spermatogenesis and testicular morphology was classified as normal based on histology, were used for Western blotting. One to two hours after surgery, pieces of chilled human tissue samples were cut, frozen in liquid nitrogen and stored at -80°C.

### Animals and ethics

The animals were obtained, housed and sacrificed according to government principles regarding the care and use of animals with permission (case number G8151/591-00.33 and JLU-419_M) of the local regulatory authority Regierungspräsidium Giessen. Rats were obtained from Janvier (Genest Saint Isle, France). Mice were purchased from Charles River Laboratories, Sulzfeld, Germany). The animals were housed at Justus-Liebig-University laboratory animal facility and maintained in a temperature controlled room with a 12 h light/dark cycle with free access to food and water. Ten adult male Wistar rats (300–330 g / approx. 3 month old) and ten adult male C57/Bl6 mice (10–12 weeks old) were used for sample collection. Mice were sacrificed by decapitation and rats were anesthetized with 5% isoflurane prior to sacrifice by cervical dislocation before dissection and collection of whole testes and lungs. Western blot samples were frozen in liquid nitrogen and stored at -80°C until protein preparations. For histological analysis, specimens were treated as described for human biopsies above.

### Western blot analysis of tight junctions

Frozen testicular and pulmonary tissue of mouse, rat and human were homogenized by ten strokes in a Potter–Elvehjem homogenizer (Wheaton, Millville, NJ, USA) and resolved in lysis buffer (20 mM Tris, pH 7.5, 150 mM NaCl, 1 mM EDTA, 1 mM EGTA, 1% Triton X-100). Samples were centrifuged at 3000 g for 8 min at 4°C to remove cell debris and nuclei. The supernatant fractions were ultra-centrifuged for 30 min at 100 000 g at 4°C. The resulting supernatant represents the cytosolic fraction and was used as control. The resulting pellets, representing the membrane fraction, were resuspended in 50 mM Tris-buffer, pH 7.5, and stored at -80°C. Protein concentrations were determined by using a dye-binding assay (Bio-Rad, Munich, Germany) according to the manufacturer’s protocol with bovine serum albumin (fraction V, Sigma–Aldrich, St. Louis, MO, USA) as standard. 50 μg of proteins were resolved by 12.5%-acrylamide SDS–PAGE under reducing conditions and transferred to nitrocellulose membranes. After staining with Ponceau S (Sigma–Aldrich) to visualize the positions of co-migrated reference proteins (Sigma–Aldrich, catalog # S8320) and pre-treatment with blocking solution (Roche, Mannheim, Germany, catalog # 1096176), blots were exposed to Claudin-3, Claudin-11 and Occludin antibodies, respectively (all purchased from Invitrogen, Camarillo, CA, USA, host: rabbit, dilution 1:1000, catalog # 34–1700, # 36–4500 and # 71–1500). Goat anti-rabbit IgG (Pierce, Rockford, IL, USA, dilution 1:2000, catalog # 31460), linked to peroxidase, served as secondary antibody. Immunoreactive bands were detected using enhanced chemiluminescence (Amersham, Braunschweig, Germany, catalog # RPN 2105) on Fuji X-ray films, type 13862 C. Exposure time was 2–10 min for tight junction proteins and 10 sec for vinculin. To confirm negative results, extended exposure (>1h) was performed. For stripping of bound antibodies, blots were incubated for 30min each in an aqueous solution of 5% (w/v) milk powder (Roth, Karlsruhe, Germany) and in 0.5 M NaCl / 0.5 M acetic acid prior to a final incubation for 2 min in 1.5 M Tris–HCl, pH 7.5. The blots were washed in water between and after these treatments. After stripping, blots were re-exposed to anti-vinculin (Sigma–Aldrich, host: mouse, 1:6000, catalog # V9264) and secondary antibody Goat anti-mouse IgG (Pierce, dilution 1:2000, catalog # 31432).

### Immunohistochemistry

Immunohistochemistry of 5 μm sections of bouin-fixed, paraffin-embedded specimen was performed comparably as described previously [21]. Claudin-11 antibody (Invitrogen) was used in a dilution of 1:400. The Envision System from DAKO (Hamburg, Germany) was used according to the manufacturer‘s instructions combined with DAB staining and counterstaining with hematoxylin. Digital images were captured by Axioskop (Carl Zeiss, Wetzlar, Germany) using software AxioVision (Carl Zeiss) and processed with Adobe Photoshop CS4 (Adobe, San Jose, CA, USA).

### Quantification and Statistical Methods

The quantitative analysis of claudin-11 staining were done and values were obtained from typically eight tubular cross-sections per specimen from all individuals of each group, representing NSP, Hyp, MA, SCO and SGA. Claudin-11 patches were categorized whether they have contact with the basal membrane or not. Values from each specimen were used for calculation of the means and their respective standard errors of the mean (SEM). The non-parametric Mann-Whitney-test (GraphPad Instat 3, GraphPad Software Inc., San Diego, CA, USA) was used to analyze differences between the experimental groups.

## Results

**Fig 1:**
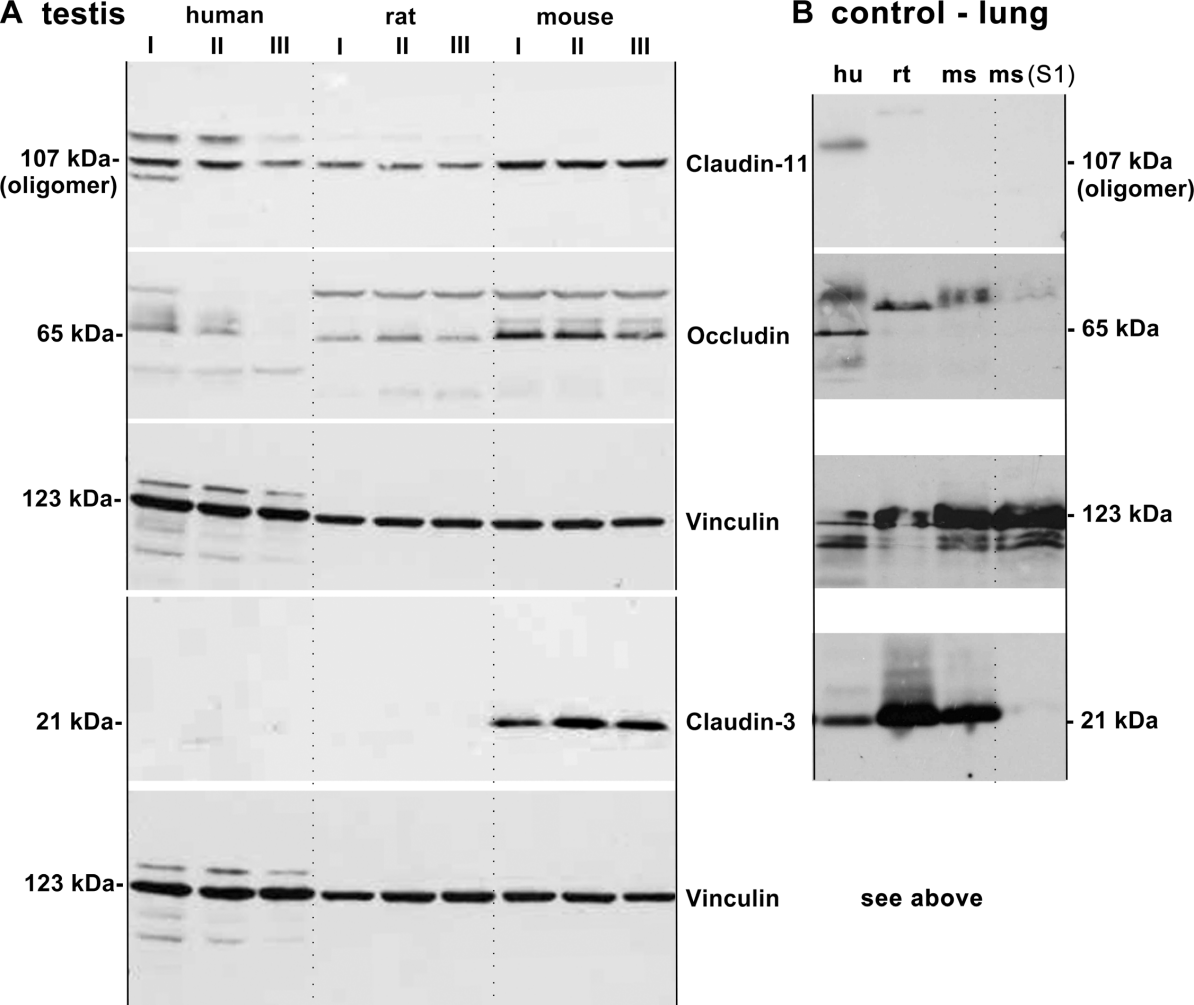
**Western Blot analysis of tight junction proteins in membrane fractions of (A) testis and (B) lung (control).** For negative controls, a soluble fraction (S1) of lung was used. (A) Tight junctions in the membrane fractions of human, mouse, and rat testis with three individuals each (I-III). The vinculin loading controls correspond to the experiments presented above, respectively. The testis of human (aged 65, 77 and 31), mouse and rat contain detectable amounts of claudin-11. On the same (stripped and retreated) membrane, occludin could only be detected clearly in mouse and rat whereas human testis contains only negligible amounts. No claudin-3 was found in testis of human and rat, but in mouse. (B) Tight junctions in the membrane fractions of human, mouse and rat lung. Vinculin loading control corresponds to all experiments. No claudin-11 was detected in the lung of rat and mouse. Occludin was found in lung of human, but not in rat or mouse. Strong claudin-3 expression was found in the lung of all three species. Mouse lung cytosolic fraction was only positive for vinculin, as expected. Abbreviations: S1 soluble fraction, hu human, ms mouse, rt rat.

Claudin-3, -11 and occludin were analyzed by immunoblotting in membrane fractions of human, mice and rat, of three individuals of each species (Fig 1A). Testis of mice showed high amounts of claudin-3, whereas in human and in rat no claudin-3 signal was detected, also not after extended exposure (>1h). The positive control (lung tissue) confirmed that human claudin-3 is detected by the antibody (Fig 1B). Occludin was found in the testis of mouse and rat, but only negligible amounts were found in human testis. Claudin-11 was detected in the testis of all three species as an oligomer, which has been described previously as a phenomenon occurring only in vitro (Invitrogen, manufacturer data sheet & pers. comm.), and as reported for other claudins [22–24]. Entire Western blot procedure was performed multiple times for each antibody and tissue with concordant results.

**Fig 2:**
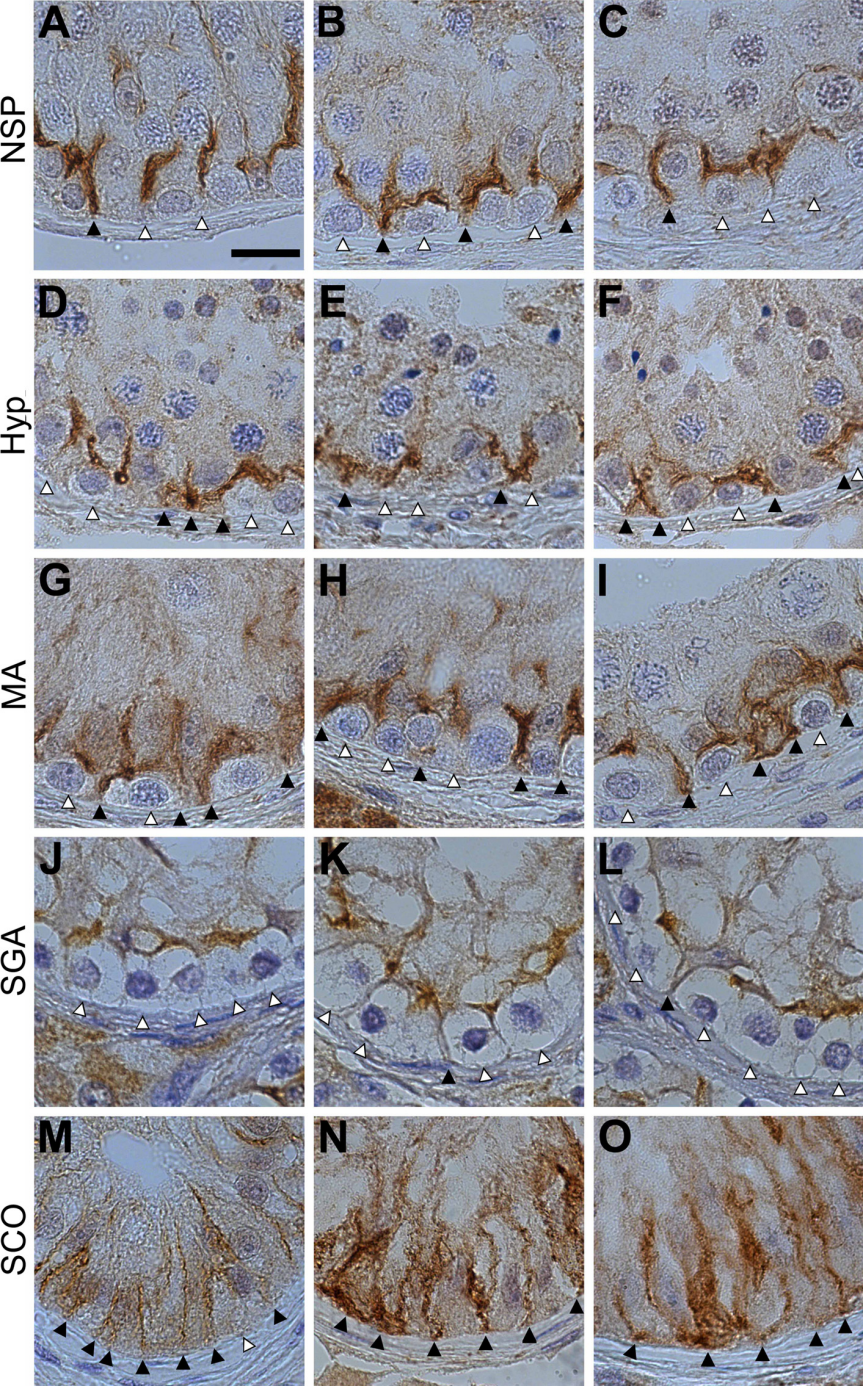
**Location of claudin-11 in human testis with normal (NSP, A-C) and impaired spermatogenesis (Hyp, D-F; MA, G-I; SGA, J-L and SCO, M-O).** Patches that have contact with the basal membrane are indicated by black arrowheads. Patches that do not touch the basal membrane are indicated by white arrowheads. The scale bar (15 μm) in image (A) applies to all images in panel I.

IHC detection of claudin-11 in the seminiferous epithelium of human, mouse, and rat produced a strong, robust staining. Claudin-11 and was selected for further analysis of impaired spermatogenesis in human testis. In human, Claudin-11 is localized at Sertoli-Sertoli junctions as well in Sertoli cells at the contacts towards spermatogonia (Fig 2, larger field view S1 Fig). The adluminal located Sertoli-Sertoli claudin-11 junctions are disrupted by migrating spermatocytes crossing the BTB (Fig 2A). Claudin-11 junctions between Sertoli cells and spermatogonia typically do not reach the basal membrane, unless flanked by the Sertoli cell body or between two Sertoli cell bodies (Fig 2B). This pattern typically occurs after spermatocytes have left the basal compartment. Dislocated spermatogonia, sloughed from the basal membrane decribed by [3] are shown by our study to rest below the barrier (Fig 2C). In testis of Hyp patients, contacts between claudin-11 patches and the basal membrane can also be found in places where no developing spermatocytes are present (Fig 2D–2F). In testis of patients diagnosed with MA, almost all spermatogonia are flanked by Sertoli cells forming claudin-11 patches that reach the basal membrane (Fig 2I), whereas in SGA patients claudin-11 patches adjacent to spermatogonia were rarely found (Fig 2J–2L). In SCOs, the frequent Sertoli-Sertoli junctions are in contact to the basal membrane (Fig 2M–2O).

**Fig 3:**
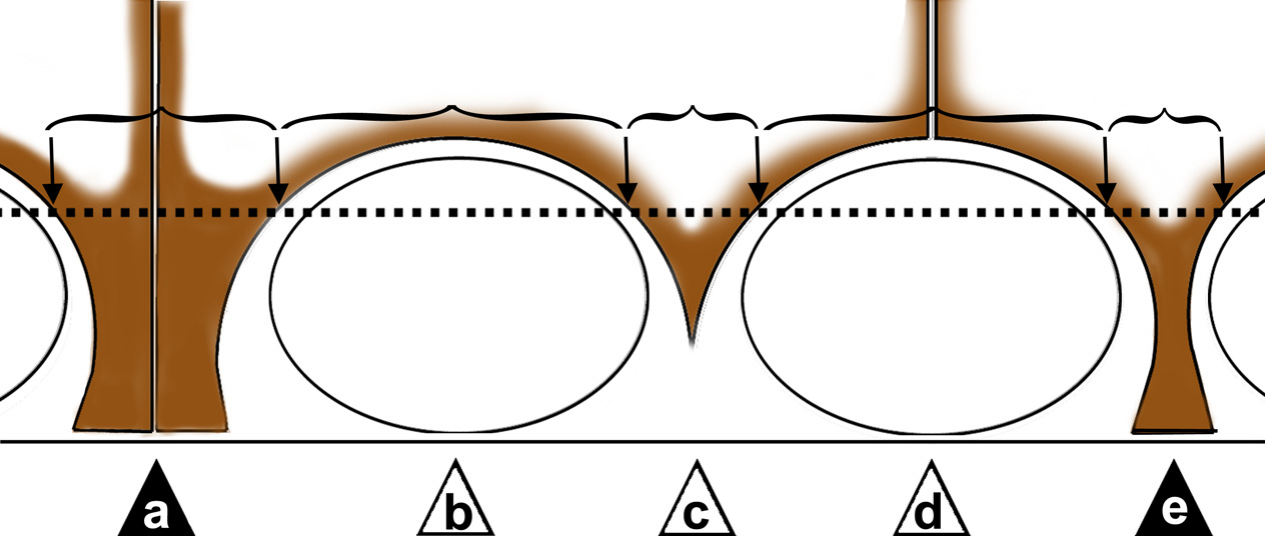
Scheme of claudin-11 expression in the seminiferous epithelium used for patch analysis. A claudin-11 patch is defined as the claudin-11 localization pattern between two points crossing the fictive line above the spermatogonia (black arrows). In the drawing the patches are indicated by brackets, letters (a-e) and triangles. If claudin-11 is in contact with the basal membrane (a & e), the patch was evaluated as positive (filled triangles), all other types negative (b-d, white triangles).

Fig 3 gives an overview over the appearance of claudin-11 patches in tubular cross sections: Sertoli-Sertoli junctions with contact to the basal membrane (a), claudin-11 shielding spermatogonia (b), claudin-11 between spermatogonia (c), Sertoli-Sertoli cell body junctions shielding spermatogonia (d). Type b-d has no contact with the basal membrane, while Sertoli cell bodies (single, type e; or paired, type a) are connected with the basal membrane.

**Fig 4:**
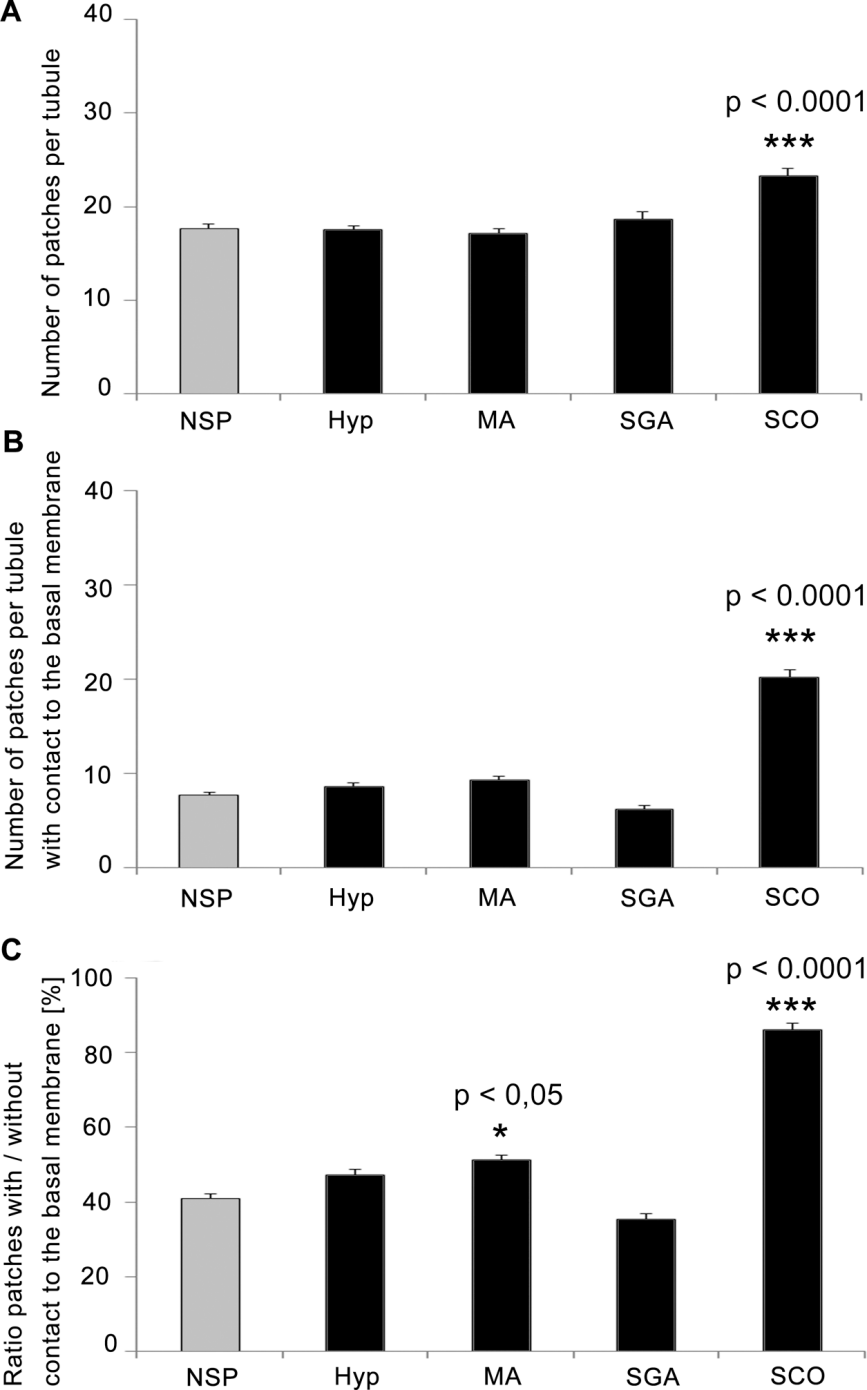
Quantitative analysis of claudin-11 localization in contact with the basal membrane in men with impaired spermatogenesis compared to normal spermatogenesis. (A) The total number of patches per tubule as well as (B) the number of patches reaching the basal membrane differs highly significant between NSP and SCO. (C) The ratio of the total number of patches versus patches with contact to the basal membrane. The ratio is significantly higher for MA and SCO. Analysis is based on a cohort of 100 patients (NSP n = 25, Hyp n = 24, MA n = 24, SCO n = 19, and SGA n = 8). Columns represent mean values with SEM indicated. Kruskal-Wallis-test was used to calculate p-values, p ≤ 0.05 was considered significant (*), p ≤ 0.001 highly significant (**), respectively, p ≤ 0.0001 (***).

In order to quantify morphological differences between the groups of patients, the numbers of claudin-11 patches per tubular cross-sections were counted in all groups (Fig 4 and S1 Table). For SCO the total number of patches (average of 24.6 patches +/- 0.770) was found to be highly significant increased (p < 0.0001) compared to NSP (17.7 +/- 0.465), (Fig 4A). The number of patches with BM-contact was also found to be highly significant increased for SCO (20.2 +/- 0.883) compared to NSP (7.7 +/- 0.332), (Fig 4B). The ratio of patches with BM-contact versus no BM-contact was calculated per cross section and compared among the groups (Fig 4C). In NSP, the ratio was about 41.0% (+/- 1.236) and 47.3% (+/- 1.502) in Hyp (not significant). Compared to NSP, the ratio was significantly increased for SCO (86.0%, +/- 1.867, p < 0.0001) and MA (51.3%, +/- 1.208, p = 0.0013), while in SGA (35.3%, +/- 1.658) the ratio was decreased, but not significantly (p = 0.0891).

## Discussion

We found claudin-11 in the seminiferous epithelium of mouse, rat and human highly conserved in Sertoli-Sertoli- and Sertoli-spermatogonia junctions, even in impaired spermatogenesis. A higher number of contacts are observed in MA patients. In SCOs, the majority of claudin-11 patches are connected with the basal membrane, while in SGA, a possible trend towards fewer basal patches was found not to be statistically significant. Basal Sertoli-Sertoli junctions typically occur in places where germ cells have left the basal compartment. The number of claudin-11 contacts with the basal membrane is rare in tubular cross-sections in NSP because in healthy spermatogenesis this gap is rapidly filled with new spermatogonia derived from spermatogonial stem cells. Thus, an increase of claudin-11 basal membrane contacts occurred concurrent with impaired spermatogenesis, while leaving the question open, whether the impairment of germ cell development is the reason or a consequence of the altered structure in the BTB.

The pattern of claudin-11 in impaired spermatogenesis was also studied by other groups [17, 25, 26] albeit with a restricted number of patients. None of the referred studies included claudin-3 into their histological analysis, whose function in BTB is intensively discussed in the mouse model [11, 27]. In line with our results, [17] described claudin-11 expression in MA patients to be shifted towards the periphery. However, the absence of claudin-11 protein in some patients with Hyp, MA and SCO as reported by [25] was not observed in our study, possibly due to a more sensitive detection method. Similarly to [26] we also found an increased adluminal localization of claudin-11, however, in our biopsies, detached Sertoli cells in the lumen were not evaluated. We analyzed claudin-11 in SGA patients (which has never been done before) and found that the claudin-11 pattern is altered coincident with the altered morphology. Claudin-11 is unique in the seminiferous epithelium and in oligodendrocytes [12, 28] and is indispensable in mice for the integrity of the seminiferous epithelium. Claudin-11 knockout mice exhibit degeneration of the seminiferous epithelium with detachment of Sertoli cells and absence of spermatogenesis [28, 29].

Claudin-3 was pointed out as a stage-specific basal barrier in mice [6, 9, 10]. In Claudin-3 KO mice, prolonged preleptotene stage was observed, suggesting basal claudin-3 expression may support the migration of spermatocytes [11]. However, claudin-3 knockout mice are fertile and have an intact BTB [27, 30]. A functional basal component of BTB was also found by tracer diffusion experiments in rat seminiferous epithelium [8], even though it is devoid of claudin-3 according to our experiments and findings by [19]. It remains to be investigated how a basal barrier is built in the absence of claudin-3 from Sertoli cells. Furthermore, our experiments verified occludin expression in seminiferous epithelium of mouse and rat and its absence from human testis, in concordance with the literature [4]. Occludin knockout mice suffer from testicular atrophy with progression of age [31]. Occludin is essential for the maintenance of the seminiferous epithelium in mice but is dispensable in humans, indicating considerable differences of the BTB among species. The essential component of the BTB, which has been described in mouse testis could only partially be confirmed in rat testis and as clearly shown not in human testis. Only claudin-11 was conserved between species and showed an altered pattern in impaired spermatogenesis. We conclude that an increased number of basal Sertoli-Sertoli junctions, evident by claudin-11 patches with contact to the basal membrane, might reflect impaired spermatogenesis without answering the question, if impairment of germ cell development is causal for altered structure in the BTB or vice versa. In patients treated 8 weeks with gonadotropin suppression, claudin-11 localization was found to be markedly disrupted, which is associated with the extent of suppression of meiosis [32]. These findings suggest that in human localization of claudin-11 might be regulated by developing germ cells as described for the basal component of the BTB represented by claudin-3 and claudin-5 in the mouse model [6, 9]. Tracer diffusion experiments with patients with impaired spermatogenesis suggest that disruption of the BTB may occur only in tubules with immature Sertoli cells [33]. Of note for further investigation, the composition of the basal component of the BTB in human testis seems to be principally different from rodent animals.

## Supporting Information

S1 Fig Location of claudin-11 in human testis with normal (NSP) and impaired spermatogenesis (Hyp, MA, SGA, and SCO,), larger field view. (TIF)

S1 Table Patients of the study: diagnosis, age, score and claudin-11 patches, the total number of patches in one tubular cross section (pat) and the number of patches in contact with the basal membrane (b pat). (PDF)
