# Supplementary figures and images for: Highly Conserved Testicular Localization of Claudin-11 in Normal and Impaired Spermatogenesis
Source: PLoS One. 2016 Aug 3;11(8):e0160349. doi: 10.1371/journal.pone.0160349 (PMC4972306; doi:10.1371/journal.pone.0160349)

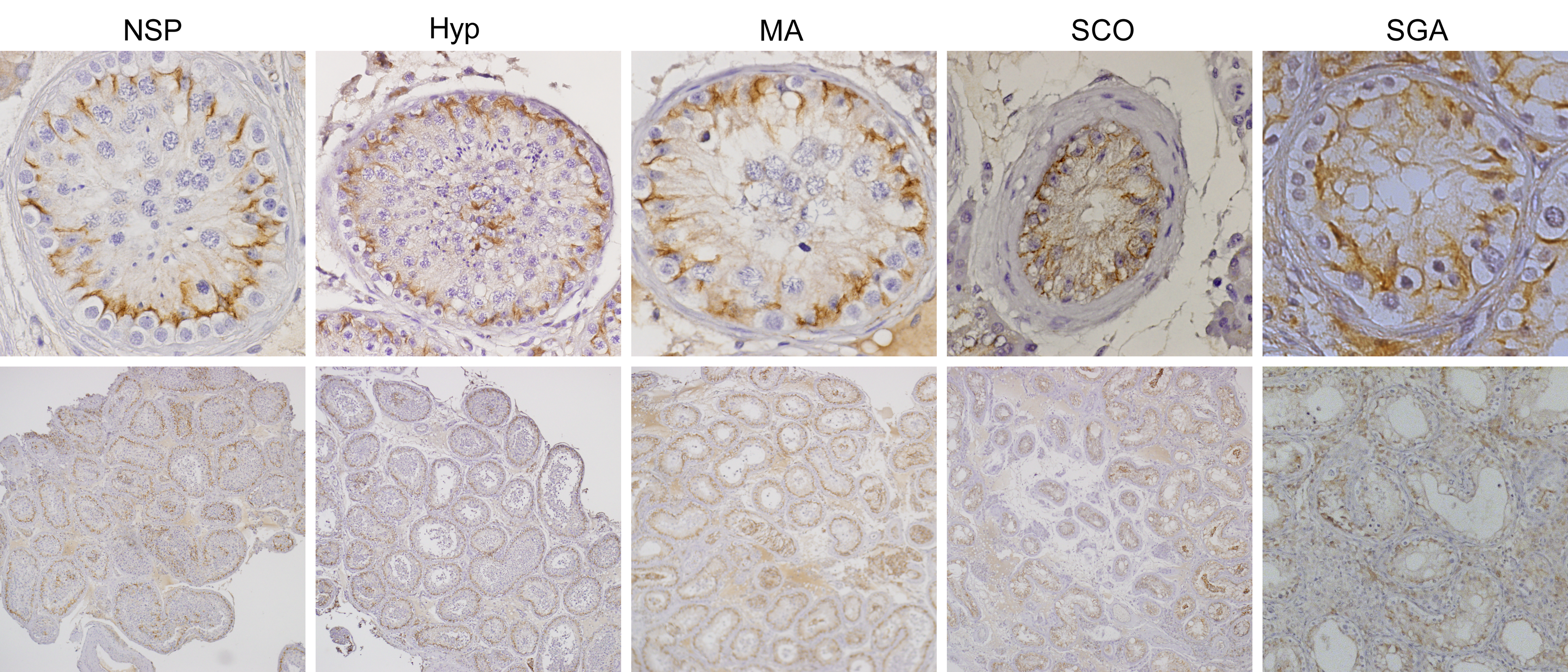

Supplement: S1 Fig — (TIF) [file pone.0160349.s001.tif]
