# Supplementary material for: Highly Conserved Testicular Localization of Claudin-11 in Normal and Impaired Spermatogenesis
Source: PLoS One. 2016 Aug 3;11(8):e0160349. doi: 10.1371/journal.pone.0160349 (PMC4972306; doi:10.1371/journal.pone.0160349)
Supplement: S1 Table — (PDF) [file pone.0160349.s002.pdf]

S1 Table. Patients of the study: diagnosis, age, score and claudin-11 patches, the total number of patches in one tubular cross section (pat) and the number of patches in contact with the basal membrane (b pat).

**NSP**	**age**	**score**	pat	b pat	ration	pat	b pat	ratio	pat	b pat	ratio	pat	b pat	ratio	pat	b pat	ratio	pat	b pat	ratio	pat	b pat	ratio	pat	b pat	ratio	mean pat	mean b pat	mean ration	SEM
	41	9	9	3	33	17	6	35	13	8	62	15	9	60	25	11	44	11	6	55	16	9	56	13	7	54	14.88	7.38	49.85	3.867
	43	10	7	1	14	10	5	50	16	8	50	17	7	41	20	6	30	20	12	60	16	7	44	18	5	28	15.50	6.38	39.62	5.218
	37	10	12	6	50	16	7	44	21	12	57	26	12	46	6	1	17	25	12	48	12	5	42	17	6	35	16.88	7.63	42.33	4.300
	32	10	21	13	62	25	18	72	25	16	64	21	11	52	30	20	67	31	14	45	25	17	68	26	12	46	25.50	15.13	59.53	3.636
	33	10	14	7	50	20	7	35	16	7	44	12	7	58	19	9	47	12	4	33	20	8	40	13	5	38	15.75	6.75	43.28	2.959
	39	10	24	14	58	29	14	48	23	9	39	36	19	53	27	11	41	22	11	50	n.d.	n.d.	n.d.	n.d.	n.d.	n.d.	26.83	13.00	48.21	2.971
	35	10	19	6	32	21	9	43	25	8	32	17	9	53	18	10	56	20	10	50	16	5	31	14	2	14	18.75	7.38	38.81	4.962
	47	10	14	3	21	17	10	59	25	9	36	32	20	63	19	10	53	17	8	47	20	8	40	11	2	18	19.38	8.75	42.08	5.782
	43	10	10	4	40	14	3	21	12	4	33	23	12	52	13	3	23	22	9	41	22	15	68	20	8	40	17.00	7.25	39.89	5.377
	37	10	22	9	41	16	5	31	15	3	20	12	4	33	24	7	29	9	4	44	14	4	29	17	6	35	16.13	5.25	32.87	2.690
	20	10	20	10	50	11	1	9	21	9	43	23	11	48	n.d.	n.d.	n.d.	n.d.	n.d.	n.d.	n.d.	n.d.	n.d.	n.d.	n.d.	n.d.	18.75	7.75	37.44	9.568
	37	9	19	14	74	14	7	50	22	11	50	10	4	40	13	3	23	11	1	9	11	2	18	9	3	33	13.63	5.63	37.17	7.359
	36	10	14	3	21	13	5	38	14	5	36	17	2	12	8	5	63	23	4	17	18	8	44	21	10	48	16.00	5.25	34.92	6.052
	38	10	12	1	8	3	0	0	5	0	0	7	1	14	4	0	0	5	0	0	1	0	0	7	2	29	5.50	0.50	6.40	3.689
	27	10	28	14	50	25	18	72	22	10	45	29	16	55	29	14	48	19	11	58	18	12	67	22	7	32	24.00	12.75	53.41	4.456
	33	10	15	5	33	26	9	35	9	6	67	21	4	19	20	10	50	15	8	53	18	11	61	20	2	10	18.00	6.88	41.01	7.120
	44	10	6	2	33	7	2	29	13	5	38	9	7	78	19	5	26	6	1	17	14	6	43	16	4	25	11.25	4.00	36.12	6.613
	40	10	20	8	40	20	12	60	30	12	40	24	12	50	25	8	32	24	13	54	11	6	55	18	11	61	21.50	10.25	48.98	3.725
	42	10	18	6	33	31	20	65	13	9	69	25	17	68	28	17	61	19	7	37	21	9	43	15	9	60	21.25	11.75	54.44	5.113
	28	10	15	5	33	9	1	11	14	5	36	9	3	33	13	6	46	22	5	23	8	3	38	15	6	40	13.13	4.25	32.48	3.855
	56	10	13	7	54	18	6	33	26	10	38	30	19	63	26	16	62	17	8	47	25	7	28	12	7	58	20.88	10.00	47.99	4.755
	40	9	20	15	75	20	9	45	28	14	50	24	18	75	21	9	43	12	6	50	22	8	36	19	9	47	20.75	11.00	52.70	5.107
	37	10	19	5	26	19	7	37	14	6	43	18	9	50	29	16	55	20	13	65	14	7	50	21	9	43	19.25	9.00	46.13	4.158
	40	10	18	10	56	15	6	40	22	3	14	24	8	33	15	9	60	12	5	42	25	12	48	14	6	43	18.13	7.38	41.88	5.050
	36	10	20	2	10	7	1	14	14	0	0	14	2	14	25	5	20	12	3	25	17	7	41	20	3	15	16.13	2.88	17.47	4.252
median	37	10																										mean ratio	41.00	1.236
mean	37.64	9.88																										mean pat	17.68	0.465
number	25																											mean b pat	7.71	0.332

abbreviations: b basal, pat patch, n.d. no data

S1 Table. Patients of the study: diagnosis, age, score and claudin-11 patches, the total number of patches in one tubular cross section (pat) and the number of patches in contact with the basal membrane (b pat).

**Hyp**	**age**	**score**	pat	b pat	ratio	pat	b pat	ratio	pat	b pat	ratio	pat	b pat	ratio	pat	b pat	ratio	pat	b pat	ratio	pat	b pat	ratio	pat	b pat	ratio	mean pat	mean b pat	mean ration	SEM
	36	10	18	17	94	12	11	92	26	23	88	23	19	83	12	11	92	15	15	100							17.67	16.00	91.47	2.375
	43	8	12	4	33	30	13	43	16	6	38	13	5	38	15	5	33	25	11	44	15	7	47	21	10	48	18.38	7.63	40.53	2.004
	39	8	9	3	33	7	3	43	7	3	43	11	5	45	11	5	45	17	9	53	13	5	38	1	0	0	9.50	4.13	37.67	5.743
	37	9	17	10	59	18	7	39	9	4	44	17	4	24	18	5	28	17	10	59	17	8	47	29	8	28	17.75	7.00	40.87	4.907
	32	8	22	14	64	26	15	58	12	7	58	10	7	70	14	10	71	14	7	50	n.d.	n.d.	n.d.	n.d.	n.d.	n.d.	16.33	10.00	61.85	3.324
	38	8	11	3	27	18	4	22	15	6	40	18	10	56	n.d.	n.d.	n.d.	n.d.	n.d.	n.d.	n.d.	n.d.	n.d.	n.d.	n.d.	n.d.	15.50	5.75	36.26	7.439
	37	10	16	7	44	27	14	52	18	9	50	20	9	45	24	12	50	17	6	35	22	11	50	26	19	73	21.25	10.88	49.87	3.818
	44	8	20	10	50	16	9	56	15	10	67	25	11	44	17	7	41	20	7	35	7	2	29	22	13	59	17.75	8.63	47.59	4.534
	35	9	26	13	50	25	9	36	27	13	48	17	13	76	24	12	50	31	18	58	18	11	61	36	23	64	25.50	14.00	55.46	4.316
	47	8	18	7	39	13	5	38	23	5	22	21	11	52	18	4	22	11	3	27	12	4	33	6	2	33	15.25	5.13	33.45	3.572
	40	9	25	9	36	16	5	31	17	3	18	22	9	41	20	8	40	14	4	29	14	5	36	13	4	31	17.63	5.88	32.61	2.639
	40	8	14	6	43	14	7	50	11	8	73	26	16	62	24	7	29	18	6	33	17	6	35	14	4	29	17.25	7.50	44.19	5.697
	28	9	22	13	59	15	9	60	23	9	39	9	2	22	18	10	56	16	8	50	11	7	64	15	8	53	16.13	8.25	50.37	4.817
	30	9	11	10	91	12	9	75	15	12	80	10	7	70	11	10	91	18	18	100	16	14	88	20	18	90	14.13	12.25	85.54	3.469
	32	7	18	10	56	17	9	53	18	8	44	29	19	66	22	11	50	26	17	65	18	12	67	28	10	36	22.00	12.00	54.53	3.932
	39	10	14	2	14	13	6	46	14	6	43	23	17	74	22	11	50	5	1	20	16	7	44	5	0	0	14.00	6.25	36.37	8.309
	40	6	16	10	63	24	15	63	18	12	67	16	5	31	16	1	6	21	16	76	18	9	50	22	12	55	18.88	10.00	51.24	7.962
	32	10	6	0	0	6	2	33	10	2	20	5	0	0	9	2	22	6	2	33	6	1	17	5	0	0	6.63	1.13	15.69	5.041
	40	9	6	0	0	10	3	30	12	3	25	10	2	20	15	7	47	16	5	31	11	3	27	19	9	47	12.38	4.00	28.44	5.335
	35	7	26	15	58	20	10	50	21	11	52	26	14	54	10	5	50	24	9	38	26	12	46	18	12	67	21.38	11.00	51.78	2.997
	44	8	24	20	83	27	16	59	13	4	31	23	15	65	13	3	23	18	8	44	22	12	55	22	10	45	20.25	11.00	50.76	6.802
	35	9	22	13	59	23	9	39	25	21	84	28	22	79	26	16	62	23	8	35	30	13	43	22	10	45	24.88	14.00	55.74	6.464
	26	9	25	16	64	16	7	44	24	9	38	26	12	46	21	9	43	13	4	31	30	13	43	25	13	52	22.50	10.38	45.05	3.482
	34	10	16	4	25	22	9	41	18	9	50	16	8	50	20	6	30	12	3	25	11	6	55	11	2	18	15.75	5.88	36.70	4.913
median	37	9																										mean ratio	47.25	1.502
mean	36.79	8.58																										mean pat	17.49	0.473
number	24																											mean b pat	8.66	0.370

abbreviations: b basal, pat patch, n.d. no data

S1 Table. Patients of the study: diagnosis, age, score and claudin-11 patches, the total number of patches in one tubular cross section (pat) and the number of patches in contact with the basal membrane (b pat).

**MA**	**age**	**score**	pat	b pat	ratio	pat	b pat	ratio	pat	b pat	ratio	pat	b pat	ratio	pat	b pat	ratio	pat	b pat	ratio	pat	b pat	ratio	mean pat	mean b pat	mean ration	SEM
	30	0	16	7	44	20	11	55	22	10	45	12	5	42	4	1	25	11	5	45	23	15	65	22	13	59	16.25	8.38	47.58	4.365
	39	0	19	7	37	17	6	35	13	3	23	13	7	54	n.d.	n.d.	n.d.	n.d.	n.d.	n.d.	n.d.	n.d.	n.d.	n.d.	n.d.	n.d.	15.50	5.75	37.26	6.327
	29	0	18	7	39	16	4	25	9	1	11	21	5	24	17	8	47	9	4	44	9	3	33	17	8	47	14.50	5.00	33.84	4.596
	32	0	10	3	30	16	12	75	7	3	43	9	4	44	16	8	50	10	5	50	12	5	42	11	4	36	11.38	5.50	46.29	4.729
	n.d.	0	15	10	67	20	12	60	18	7	39	14	6	43	25	13	52	20	11	55	9	4	44	17	11	65	17.25	9.25	53.07	3.667
	30	0	19	9	47	19	18	95	16	5	31	16	9	56	25	17	68	23	8	35	9	3	33	19	9	47	18.25	9.75	51.64	7.580
	33	0	10	4	40	18	8	44	18	6	33	11	9	82	24	12	50	16	6	38	18	7	39	12	6	50	15.88	7.25	47.00	5.393
	32	0	6	1	17	6	3	50	7	4	57	8	3	38	10	2	20	10	3	30	14	11	79	9	4	44	8.75	3.88	41.79	7.229
	22	0	20	14	70	10	2	20	12	5	42	21	14	67	15	14	93	9	5	56	13	8	62	20	13	65	15.00	9.38	59.22	7.599
	38	0	4	0	0	10	4	40	12	5	42	15	4	27	9	5	56	13	8	62	15	8	53	16	8	50	11.75	5.25	41.10	7.014
	32	0	17	12	71	13	7	54	11	7	64	11	4	36	12	4	33	5	1	20	6	4	67	8	3	38	10.38	5.25	47.74	6.521
	17	0	21	12	57	25	16	64	22	11	50	30	16	53	13	10	77	22	9	41	19	13	68	12	7	58	20.50	11.75	58.63	3.960
	47	0	25	15	60	23	20	87	25	22	88	15	6	40	13	8	62	13	8	62	9	6	67	24	18	75	18.38	12.88	67.46	5.573
	36	0	20	9	45	21	14	67	20	4	20	21	12	57	13	5	38	17	12	71	13	10	77	24	18	75	18.63	10.50	56.22	7.135
	31	0	21	12	57	10	6	60	19	10	53	17	9	53	17	14	82	12	4	33	8	6	75	15	9	60	14.88	8.75	59.18	5.248
	29	0	22	12	55	24	15	63	8	5	63	9	3	33	15	5	33	n.d.	n.d.	n.d.	n.d.	n.d.	n.d.	n.d.	n.d.	n.d.	15.60	8.00	49.24	6.655
	31	0	13	6	46	13	7	54	13	5	38	14	9	64	8	2	25	9	7	78	19	12	63	8	4	50	12.13	6.50	52.34	5.814
	30	0	24	14	58	21	15	71	5	2	40	17	10	59	14	5	36	17	7	41	13	2	15	17	5	29	16.00	7.50	43.78	6.411
	30	0	20	10	50	23	17	74	20	11	55	25	13	52	15	8	53	17	8	47	20	10	50	18	7	39	19.75	10.50	52.52	3.518
	44	0	13	9	69	22	12	55	31	18	58	19	8	42	21	10	48	34	19	56	24	11	46	11	6	55	21.88	11.63	53.48	2.986
	34	0	40	25	63	31	14	45	28	17	61	34	21	62	34	24	71	36	27	75	44	31	70	29	26	90	34.50	23.13	66.98	4.563
	32	0	23	14	61	25	16	64	21	13	62	26	11	42	32	23	72	19	11	58	17	10	59	14	7	50	22.13	13.13	58.46	3.166
	31	0	18	12	67	20	9	45	16	11	69	28	24	86	32	24	75	19	8	42	19	11	58	21	7	33	21.63	13.25	59.31	6.359
	26	0	14	7	50	13	10	77	18	10	56	23	7	30	15	10	67	21	8	38	8	3	38	11	3	27	15.38	7.25	47.81	6.257
	35	0	22	15	68	31	19	61	19	6	32	18	7	39	21	12	57	19	11	58	20	8	40	22	12	55	21.50	11.25	51.19	4.519
median	32	0																										mean ratio	51.33	1.208
mean	32.08	0.00																										mean pat	17.17	0.504
number	24																											mean b pat	9.32	0.403

abbreviations: b basal, pat patch, n.d. no data

S1 Table. Patients of the study: diagnosis, age, score and claudin-11 patches, the total number of patches in one tubular cross section (pat) and the number of patches in contact with the basal membrane (b pat).

**SCO**	**age**	**score**	pat	b pat	ratio	pat	b pat	ratio	pat	b pat	ratio	pat	b pat	ratio	pat	b pat	ratio	pat	b pat	ratio	pat	b pat	ratio	pat	b pat	ratio	mean pat	mean b pat	mean ration	SEM
	38	0	34	32	94	8	7	88	24	22	92																22.00	20.33	91.09	1.932
	31	0	31	30	97	28	28	100	18	18	100	19	18	95	30	28	93	20	19	95	25	25	100	21	18	86	24.00	23.00	95.69	1.708
	23	0	17	16	94	20	20	100	15	13	87	19	14	74	8	6	75	12	11	92	12	9	75	27	26	96	16.25	14.38	86.55	3.760
	21	0	24	17	71	34	26	76	23	18	78	18	11	61	25	22	88	35	27	77	28	23	82	44	42	95	28.88	23.25	78.68	3.679
	39	0	36	31	86	23	18	78	31	29	94	27	27	100	26	25	96	24	22	92	n.d.	n.d.	n.d.	n.d.	n.d.	n.d.	27.83	25.33	90.96	3.166
	39	0	20	18	90	29	25	86	18	15	83	21	15	71	19	15	79	26	19	73	25	21	84	23	15	65	22.63	17.88	79.03	2.985
	42	0	28	27	96	25	22	88	27	26	96	18	17	94	n.d.	n.d.	n.d.	n.d.	n.d.	n.d.	n.d.	n.d.	n.d.	n.d.	n.d.	n.d.	24.50	23.00	93.79	1.983
	36	0	27	25	93	24	21	88	30	28	93	22	20	91	26	23	88	n.d.	n.d.	n.d.	n.d.	n.d.	n.d.	n.d.	n.d.	n.d.	25.80	23.40	90.56	1.134
	36	0	13	4	31	21	7	33	14	2	14	18	7	39	20	3	15	19	7	37	17	5	29	25	8	32	18.38	5.38	28.82	3.280
	30	0	40	39	98	25	19	76	n.d.	n.d.	n.d.	n.d.	n.d.	n.d.	n.d.	n.d.	n.d.	n.d.	n.d.	n.d.	n.d.	n.d.	n.d.	n.d.	n.d.	n.d.	32.50	29.00	86.75	10.750
	40	0	30	29	97	37	32	86	27	25	93	27	26	96	38	38	100	32	31	97	n.d.	n.d.	n.d.	n.d.	n.d.	n.d.	31.83	30.17	94.82	1.924
	30	0	24	22	92	27	25	93	24	21	88	27	26	96	n.d.	n.d.	n.d.	n.d.	n.d.	n.d.	n.d.	n.d.	n.d.	n.d.	n.d.	n.d.	25.50	23.50	92.01	1.807
	33	0	39	35	90	23	20	87	32	25	78	30	26	87	20	19	95	n.d.	n.d.	n.d.	n.d.	n.d.	n.d.	n.d.	n.d.	n.d.	28.80	25.00	87.30	2.738
	36	0	31	26	84	29	27	93	28	27	96	27	23	85	n.d.	n.d.	n.d.	n.d.	n.d.	n.d.	n.d.	n.d.	n.d.	n.d.	n.d.	n.d.	28.75	25.75	89.65	3.044
	35	0	30	28	93	22	18	82	32	28	88	31	27	87	16	11	69	14	11	79	n.d.	n.d.	n.d.	n.d.	n.d.	n.d.	24.17	20.50	82.84	3.501
	41	0	14	13	93	13	13	100	n.d.	n.d.	n.d.	n.d.	n.d.	n.d.	n.d.	n.d.	n.d.	n.d.	n.d.	n.d.	n.d.	n.d.	n.d.	n.d.	n.d.	n.d.	13.50	13.00	96.43	3.571
	34	0	29	25	86	26	20	77	n.d.	n.d.	n.d.	n.d.	n.d.	n.d.	n.d.	n.d.	n.d.	n.d.	n.d.	n.d.	n.d.	n.d.	n.d.	n.d.	n.d.	n.d.	27.50	22.50	81.56	4.642
	28	0	34	31	91	29	28	97	n.d.	n.d.	n.d.	n.d.	n.d.	n.d.	n.d.	n.d.	n.d.	n.d.	n.d.	n.d.	n.d.	n.d.	n.d.	n.d.	n.d.	n.d.	31.50	29.50	93.86	2.688
	35	0	38	37	97	43	37	86	30	27	90	30	30	100	40	38	95	32	31	97	43	37	86	32	30	94	36.00	33.38	93.14	1.857
median	35	0																										mean ratio	85.98	1.867
mean	34.05	0.00																										mean pat	24.62	0.770
number	19																											mean b pat	20.19	0.883

**SGA**	**age**	**score**	pat	b pat	ratio	pat	b pat	ratio	pat	b pat	ratio	pat	b pat	ratio	pat	b pat	ratio	pat	b pat	ratio	pat	b pat	ratio	pat	b pat	ratio	mean pat	mean b pat	mean ration	SEM
	38	0	18	10	56	24	12	50	n.d.	n.d.	n.d.	n.d.	n.d.	n.d.	n.d.	n.d.	n.d.	n.d.	n.d.	n.d.	n.d.	n.d.	n.d.	n.d.	n.d.	n.d.	21.00	11.00	52.78	2.778
	31	0	12	6	50	12	6	50	11	1	9	8	2	25	12	2	17	21	8	38	18	5	28	23	2	9	14.63	4.00	28.17	5.882
	36	0	17	7	41	16	6	38	19	9	47	20	8	40	30	6	20	15	5	33	13	4	31	28	9	32	19.75	6.75	35.29	2.914
	82	0	21	7	33	21	6	29	18	8	44	13	5	38	17	5	29	13	6	46	23	10	43	17	7	41	17.88	6.75	38.13	2.437
	36	0	21	6	29	37	12	32	18	10	56	21	8	38	17	10	59	30	13	43	19	4	21	23	8	35	23.25	8.88	39.08	4.587
	37	0	24	4	17	11	2	18	34	10	29	25	10	40	26	8	31	28	11	39	20	8	40	12	3	25	22.50	7.00	29.91	3.353
	51	0	10	1	10	25	4	16	28	11	39	29	5	17	14	5	36	8	1	13	15	2	13	22	3	14	18.88	4.00	19.71	3.971
	51	0	15	7	47	8	3	38	9	3	33	14	4	29	7	3	43	17	6	35	16	8	50	16	6	38	12.75	5.00	38.97	2.513
median	38	0																										mean ratio	35.25	1.658
mean	45.25	0.00																										mean pat	18.60	0.894
number	8																											mean b pat	6.22	0.406

abbreviations: b basal, pat patch, n.d. no data
